# Pure Strontium Salts

**Published:** 1892-04

**Authors:** 


### Selections and Abstracts.

### PURE STRONTIUM SALTS.
(PARAF-JAVAL)

These salts, from which every trace of Barium has been removed, are those with which the very successful investigations were originally carried out at the Paris Hospitals and which alone are mentioned in the communications read before the French Academy of Medicine and other learned Societies.

There has always been a vaguely expressed, but generally accepted opinion, that Strontium salts participate in the poisonous properties of Barium, on account of the close approximation which the two metals hold in their chemical position to the other elements.

The new and precise investigations however of Dr. Laborde, *chef des Travaux physiologiques a la Faculte de Medecine de Paris*, have put an end to this legend; the communications made by this *savant* to the French Academy of Medicine (1) and to the Society of Biology, have established once and for all, that far from being harmful, pure Strontium salts (Paraf-Javal) have on the contrary a favorable influence on the phenomena of nutrition.

The same authority showed that the previous contradictions and errors on the subject of the toxic effects of the Strontium salts were due exclusively to the greater or less impurity of the commercial products used, containing small amounts of baryta.

Professor Germain See in affirming the absolute innocuousness and remarkable therapeutical action of the Strontium salts, (Paraf-Javal) in certain maladies, mentions the fact that they were already the subject of an inaugural thesis inspired by the late Professor Vulpian in 1885 (1).

(1) Academie de Medecine de Paris (Seances du 21 et 28 Juillet 1891.)

(1) Academie de Medecine (28 Juillet 1885).

Drs. Constantin Paul and Dujardin-Beaumetz are not less positive of the merits of the Strontium salts, (Paraf-Javal) (2).

Dr. Constantin Paul, referring to his experiments, says:—"I gave 6 grammes daily of Bromide of Strontium (Paraf-Javal) to a young girl suffering from hysterical epilepsy, for two months.

"The attacks had hitherto returned periodically before the menses and resisted the regular daily administration of 4 grammes of Bromide of Potassium.

"The Bromide of Strontium (Paraf-Javal) appears to have prevented the attacks, for they have not since recurred."

Dr. Dujardin-Beaumetz found that Bromide of Strontium (Paraf-Javal) possesses the indisputable advantage of being better borne by the stomach than the other alkaline bromides.

The important position occupied by Bromide of Potassium in the treatment of nervous diseases is well known, but unfortunately, if administered for any length of time, it provokes intolerance, which, in addition to a disturbance of general nutrition, gives rise to symptoms of intestinal septicæmia, followed by cutaneous eruptions associated with intense depression and cerebral torpor.

It is therefore eminently desirable to find a substitute, a succedaneum, to use a therapeutical term, for Bromide of Potassium, a drug in fact which shall possess all its advantages without its drawbacks.

That Bromide of Strontium responds precisely to this *desideratum*, has been already proved by the clinical experimentation made; the pure salt in crystalline needles, such as has been obtained by Paraf-Javal, such as is found in the solution prepared by Chapoteaut, is soluble in all proportions of water; it is with this salt and this alone, on account of its perfect preparation and absolute purity, that clinical researches have been brought to their present pitch of constancy and precision.

At the *seance* of the Society of Biology (Paris) the 17th

(2) Societe de Therapeutique (Seance du 11 Nov., 1891).

October, 1891, Dr. Ch. ere in reporting the results observed in his hospital practice t Bicetre (1), referred to the interesting case of a patient treated with 10 grammes of Bromide of Potassium daily, in whom the cutaneous eruption persisted in spite of intestinal asepsis. This patient was given the same dose of Bromide of Strontium (Paraf-Javal) and equally good effects were obtained therapeutically without any undesirable symptoms. Intravenous injections in rabbits have shown that these animals support .85 grammes of Bromide of Strontium as against .14 of Bromide of Potassium. This proves that Bromide of Strontium (Paraf-Javal) is six times better tolerated than Bromide of Potassium.

Professor Germain See says of pure Bromide of Strontium (Paraf-Javal) that "it never produces any disastrous effect on the stomach even in large doses. It may be taken in doses of 4 grammes (62 grains) at each of the three daily meals. Out of 32 patients suffering from gastric dilatation, several have been improved and some altogether cured. I believe that the Bromide of Strontium (Paraf-Javal) will advantageously take the place of Bromide of Potassium, and especially the polybromides in the treatment of Epilepsy." (Academie de Medecine, Octobre, 1891.)

The abundant evidence at our disposal proves that the pure Bromide of Strontium. (Paraf-Javal) responds to the same indications as Bromide of Potassium, over which it has the immense advantage of being admirably tolerated; for even in large doses, it produces no accidents.

(1) Comptes-rendus de la Societe de Biologie (p. 665).

---

Dr. L. D. McIntosh, Vice-President of McIntosh Battery and Optical Company, of Chicago, died very suddenly on Tuesday, March 1st, at De Funiak Springs, Florida, where he had gone to lecture before the Florida Chautauqua, on microscopy and kindred subjects.

